# Teratogenic Effects of Organohalogen Contaminants Extracted from Whale Bacon in a Whole-Rat-Embryo Culture System

**DOI:** 10.3390/ijerph191912065

**Published:** 2022-09-23

**Authors:** Masaharu Akita, Osamu Kimura, Kazutaka Atobe, Tetsuya Endo, Shizuo Yamada, Koichi Haraguchi, Yoshihisa Kato

**Affiliations:** 1 Faculty of Family and Consumer Sciences, Kamakura Women’s University, Kamakura 247-8511, Japan; 2 School of Pharmaceutical Sciences, Health Science University of Hokkaido, Tobetsu 061-0293, Japan; 3 Kagawa School of Pharmaceutical Sciences, Tokushima Bunri University, Sanuki 769-2193, Japan; 4 Center for Pharma-Food Research (CPFR), Graduate School of Pharmaceutical Sciences, University of Shizuoka, Shizuoka 422-8526, Japan; 5 Department of Pharmaceutical Sciences, Daiichi University of Pharmacy, Fukuoka 815-8511, Japan

**Keywords:** morphological abnormality, polychlorinated biphenyl, rat embryo, whale bacon, whole-embryo culture

## Abstract

Marine foods can be contaminated with organochlorines and the risk to human beings who consume these foods needs to be evaluated. We examined the teratogenic effects of contaminants extracted from whale bacon on rat embryos using a whole-embryo culture system. Embryonic day 11.5 embryos were cultured for 48 h with organohalogens extracted from whale bacon at low (polychlorinated biphenyls (PCBs): 0.32 ppm, dichlorodiphenyltrichloroethanes (DDTs): 0.16 ppm, chlordanes (CHLs): 0.02 ppm) and high (PCBs: 2.15 ppm, DDTs: 1.99 ppm, CHLs: 0.20 ppm) doses. The levels of organohalogen compounds in cultured embryos were determined. The organochlorine contaminants extracted from whale products were readily transferred to the cultured rat embryos. The number of heartbeats, yolk sac circulation score, and embryonic body circulation score of embryos did not change during the culture period in either exposure group. Cultured embryos treated with the low-dose contaminated medium for 48 h showed abnormalities of the mandible, and craniofacial or forelimb hematomas with an incidence of 50%. All embryos treated with the high-dose medium showed craniofacial abnormalities and cleft lip, and limb abnormalities and hematomas. These results indicate that the organohalogen contaminants in whale bacon may be teratogenic in a dose-dependent manner. Further studies are necessary to determine the dose–effect relationship.

## 1. Introduction

Organohalogen compounds, such as polychlorinated biphenyls (PCBs), dichlorodiphenyltrichloroethanes (DDTs), and chlordanes (CHLs), are widely distributed throughout the environment, and they readily accumulate in the marine food chain, especially in odontoceti cetaceans, such as toothed whales and dolphins [1]. Small cetaceans have traditionally been hunted in Japanese coastal waters, and cooked, raw, or frozen meat and blubber products have been consumed [2]. We have surveyed the contaminant levels of organochlorines and heavy metals in whale meat products marketed for human consumption in Japan [2,3] and lipid-rich products of cetaceans are highly contaminated with organochlorines [4]. In addition, in aquatic animals such as fish, these contaminants have been detected at lower concentrations [5,6].

These products have the potential to impair development and to cause adverse health effects on the reproductive systems of human beings and animals [1]. There is a need to evaluate the risk to human beings who consume such foods. Thus, environmentally relevant PCB congeners have been investigated for cardiac development in the chick embryo [7] and zebrafish embryo [8]. The whole-embryo culture system is an excellent method to screen drugs and organohalogen compounds for teratogenic effects [9,10], and teratogenic studies on some polychlorinated compounds have been performed in rat whole-embryo systems [11]. However, investigation of the embryonic effects of organohalogen mixtures extracted from whale products has been limited.

In the present study, we chose two exposure doses of the organohalogen mixtures extracted from whale bacon and examined the teratogenic effects of the contaminants extracted from whale bacon on embryonic day 11.5 rat embryos cultured for 48 h in a whole-rat-embryo culture system.

## 2. Materials and Methods

### 2.1. Contaminant Extraction from Whale Bacon Products

The whale bacon used in this study was purchased from a retail outlet in Fukuoka in 2000. The species of origin was identified as pilot whale (*Globicephala macrorhynchus*) by DNA analysis [12]. This product has been commonly sold for human consumption and the contamination patterns in 2000 are almost the same as those in whale blubber during the recent two decades, although the concentrations have been decreasing [4,13]. The organohalogen PCB, DDT and CHL contaminants were extracted and purified using the gel permeation chromatography and silica gel column method of Haraguchi et al. [14]. The lipid extraction rate was 55.1%. The extracts were dissolved in dimethyl sulfoxide (DMSO) (Wako Pure Chemical Industries, Ltd., Osaka, Japan).

### 2.2. Determination of Organohalogens in Whale Bacon

Chemicals were identified using a GC/MS system (GC-17A, QP-5000, Shimadzu, Japan) with a DB-5 capillary column (60 m × 0.25 mm, i.d.). Temperature program: 100 °C, 2 min, 100–250 °C at 20 °C/min, 250–280 °C at 2 °C/min [15]. ΣPCB: sum of 14 PCB isomers, ΣDDT: sum of *p*,*p*′-DDE [1,1-bis(4-chlorophenyl)-2,2-dichloroethylene], *p,p*′-DDT [1,1-bis(4-chlorophenyl)-2,2,2-trichloroethane], and *p*,*p*′-DDD [1,1-bis(4-chlorophenyl)-2,2-dichloroethane], ΣCHL: sum of trans-/cis-nonachlor, trans-/cis-chlordane, and oxychlordane (Supplementary Table S1).

### 2.3. Ethics Statement

All experimental procedures were carried out in accordance with the approved guidelines of the Institutional Animal Care and Use Committee of Kamakura Women’s University (Kamakura, Japan). The study was approved by the Research Ethics Committee and the Experiment Animal Committee of Kamakura Women’s University.

### 2.4. Whole-Embryo Culture

The whole-embryo culture system (Model: 10-1-0310, Ikemoto Rika, Tokyo, Japan) was used according to the method of New [9] and New and Cockroft [10] (Supplementary Figure S1). We chose the culture stage and time as the period when organohalogen-contaminated extracts manifest their effects on in vivo limb formation. In preliminary experiments, we determined the optimal conditions for the chosen time of embryo culture. Embryos were removed at embryonic day 11.5 (E11.5). The uterus from pregnant rats was transferred to a dish of saline solution and carefully torn open with forceps. This exposed the pear-shaped masses of decidual tissue from which the embryos and their membranes can be dissected out under low-power magnification. The outermost membrane layer, Reichert’s membrane, with attached trophoblast and parietal endoderm, was opened but the visceral yolk sac, amnion and ectoplacental cone were left intact and were explanted with the embryo into Tyrode’s solution (0.8% NaCl, 0.02% KCl, 0.02% CaCl_2_, 0.01% MgCl_2_, 0.005% NaH_2_PO_4_, 0.1% NaHCO_3_, 0.1% glucose). The explanted embryos were then placed in the nutrient medium in a gas-tight chamber containing a suitable oxygen and carbon dioxide mixture (Supplementary Figure S1). After incubation for 2 h, the organohalogen-contaminated extracts dissolved in DMSO (10 µL) were added to the medium (final concentrations: 0.32 ppm for PCBs, 0.16 ppm for DDTs, and 0.02 ppm for CHLs for low-dose exposure, and 2.15 ppm for PCBs, 1.99 ppm for DDTs, and 0.20 ppm for CHLs for high-dose exposure) (Table 1). The control embryos were cultured in the culture medium with or without vehicle (DMSO). The E11.5 embryos were cultured for 48 h at 38 °C with 95% O_2_ and 5% CO_2_ by rotating in 100% rat serum prepared immediately after exsanguination from male Wistar–Imamichi rats (weighing 200–230 g, Institute for Animal Reproduction, Kasumigaura, Japan).

The number of heartbeats, yolk sac circulation rates and embryonic blood circulation rates of embryos were measured at 2, 4, 24 and 48 h after initiating culture. Embryos were examined for morphology, crown–rump length and total number of somites after culture for 24 and 48 h as described previously [9,10].

### 2.5. Determination of Organohalogens in the Culture Medium and Embryos

**Figure 1:**
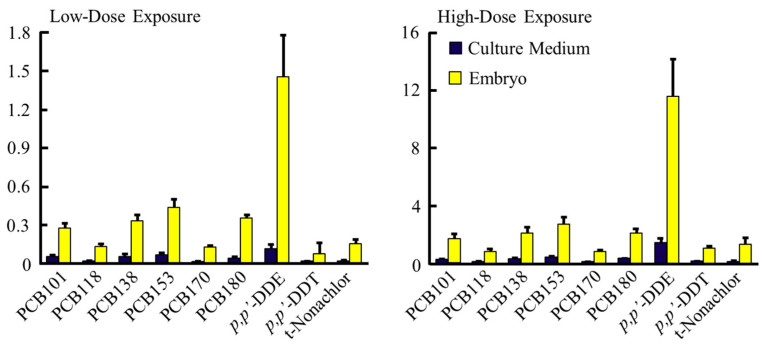
Major congener profiles of organohalogens in the culture medium and embryos with low- and high-dose exposure. E11.5 rat embryos were cultured for 48 h at 38 °C by rotating in 100% rat serum. Each column represents the mean ± S.D. (vertical bars) for four embryos. PCB 101, 2,2′,4,5,5′-pentachlorobiphenyl; PCB 118, 2,3′,4,4′,5-pentachlorobiphenyl; PCB 138, 2,2′,3,4,4′,5′-hexachlorobiphenyl; PCB 153, 2,2′,4,4′,5,5′-hexachlorobiphenyl; PCB 170, 2,2′,3,3′,4,4′,5-heptachlorobiphenyl; PCB 180, 2,2′,3,4,4′,5,5′-heptachlorobiphenyl; *p,p′*-DDE, 1,1-bis(4-chlorophenyl)-2,2-dichloroethylene; *p,p′*-DDT, 1,1-bis(4-chlorophenyl)-2,2,2-trichloroethane; t-Nonachlor, trans-nonachlor.

The culture medium and embryos were homogenized with methanol/water (1:1) and the organohalogens were extracted by n-hexane, then were purified by silicagel column chromatography. The levels of organohalogens in the culture medium and embryos were determined by GC/MS as described previously [15]. Major congeners in the medium and embryos were PCB101, 118, 138, 153, 170, and 180 for PCBs, *p*,*p*′-DDE and *p*,*p*′-DDT for DDTs and trans-nonachlor for CHLs, at low- and high-dose exposures (Figure 1).

### 2.6. Statistics

The data obtained were statistically analyzed using Student’s *t* test after analysis of variance by the Shapiro–Wilk test (SPSS, IBM). The results in Figure 1 and Table 1, Table 2 and Table 3 are the mean ± standard deviation (S.D.).

## 3. Results and Discussion

The whole-embryo culture system described by New [9] and New and Cockroft [10] is an excellent method to screen drugs and organohalogen compounds for teratogenic effects. Therefore, we used this system to investigate the direct effects of the organohalogen contaminants during the critical period of organogenesis in rat embryos. We selected two exposure levels of 0.3 and 2 ppm of PCB mixture (low and high doses, respectively) for the teratogenic study. According to recent investigation, the human dietary exposure to PCB is estimated to be among 17 ng/day [16] and around 2 pg/g in human serum [17]. In the present embryo system, we prepared two kinds of exposure medium, setting them at two or three orders of magnitude higher than human exposure levels.

The levels of the organohalogen compounds in the culture medium and embryos, and ratios of compounds in the culture medium to embryos, are shown in Table 1 and Figure 1. After incubation of E11.5 embryos for 48 h, the levels of total PCBs, total DDTs, and total CHLs in embryos were 2.44, 1.48, and 0.17 ppm, respectively, for low-dose exposure, and 13.4, 15.7, and 1.74 ppm, respectively, for high-dose exposure. The transfer ratios of the contaminants to the rat embryos from the culture medium were 7.6 and 6.2 for PCBs, 9.3 and 7.9 for DDTs and 8.5 and 8.7 for CHLs for low- and high-dose media, respectively. The results show that the organochlorine contaminants extracted from whale products were readily transferred to rat embryos in the whole-rat-embryo culture system.

**Figure 2:**
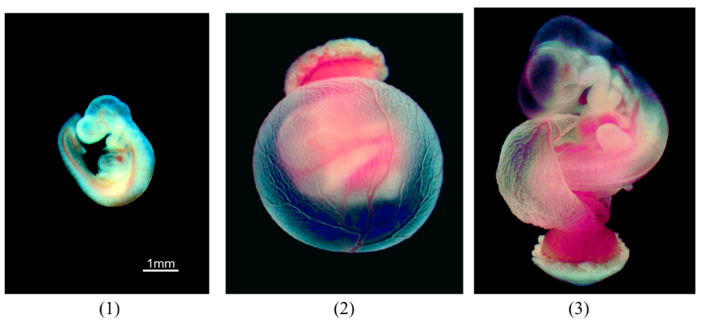
Morphological changes of rat embryos cultured in the control medium. (**1**) Whole rat embryo without the placenta and yolk sac isolated from the uterus (incubation time = 0). (**2**) Whole rat embryo with the placenta and yolk sac cultured for 24 h. (**3**) Whole rat embryo with the placenta and yolk sac cultured for 48 h. Scale bar = 1 mm.

**Figure 3:**
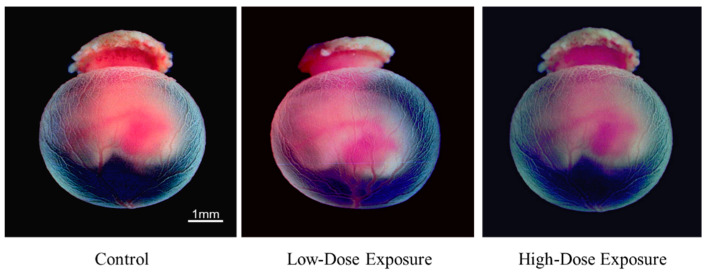
Morphological changes in rat embryos cultured for 24 h with low- and high-dose organohalogen contaminants from whale bacon. Whole cultured rat embryos with placenta and yolk sac. Scale bar = 1 mm.

**Figure 4:**
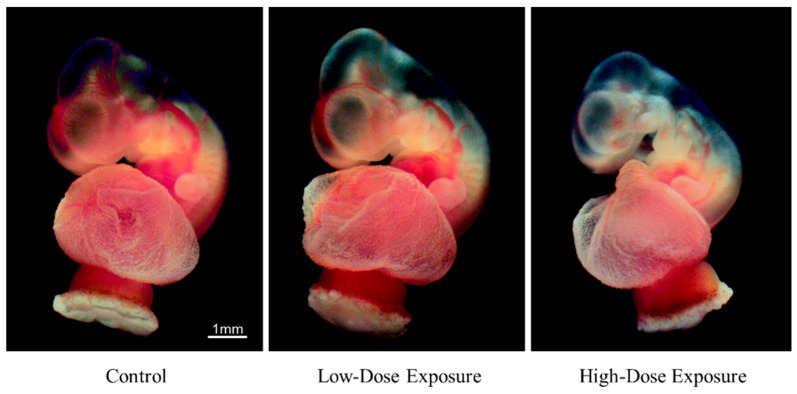
Morphological changes in rat embryos cultured for 48 h with low- and high-dose organohalogen contaminants from whale bacon. Whole cultured rat embryos with placenta and yolk sac. Scale bar = 1 mm.

**Figure 5:**
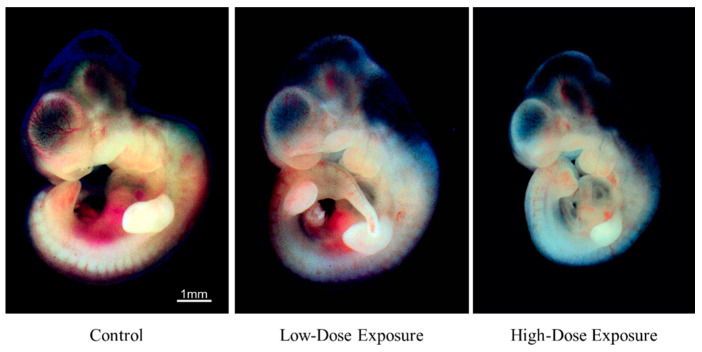
Morphological changes in rat embryos cultured for 48 h with low- and high-dose organohalogen contaminants from whale bacon. Whole cultured rat embryos without placenta and yolk sac. Scale bar = 1 mm.

The morphological changes of the rat embryos cultured in the control medium are shown in Figure 2 and Table 2. Figure 3 and Figure 4 show the embryos with placenta and yolk sac at 24 and 48 h, respectively, and Figure 5 shows the embryos without placenta and yolk sac at 48 h after initiation of incubation with the organohalogen contaminants. After incubation for 24 and 48 h, there were no differences in the number of heartbeats, crown–rump length, total number of somites, yolk sac circulation score, or embryonic body circulation score of rat embryos in control groups without and with vehicle (Table 2 and Table 3). The body size of the embryos after culture for 48 h was larger than that of the E11.5 rat embryos (before culture) (Figure 2 and Figure 5). The number of heartbeats was maintained at about 180 beats/min in all groups. After culture for 48 h, the crown–rump length and total number of somites were higher than those of the E11.5 embryos (Figure 2 and Figure 5 and Table 2). These results indicate that the cultured embryos grew in a manner similar to embryos in utero.

Compared with the vehicle control group, the number of heartbeats did not change during the culture period in either exposure group. Embryos with an abnormal yolk sac circulation score or an embryonic body circulation score were not detected in either exposure group during the culture period (Table 3). However, in the high-dose exposure group, the crown–rump length and total number of somites after incubation for 48 h were lower than those of the vehicle control group (Table 2 and Figure 5).

**Figure 6:**
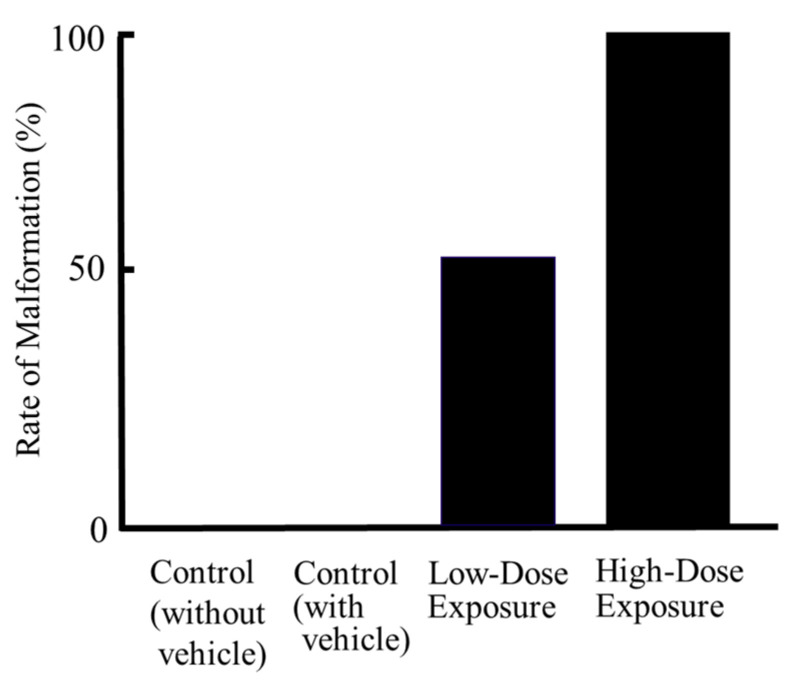
The rates of morphogenic abnormalities in rat embryos (n = 12) cultured for 48 h with low- and high-dose organohalogen contaminants from whale bacon. Number of embryos: control (without vehicle), 8; control (with vehicle), 8; low-dose exposure, 16; high-dose exposure, 17. The morphogenic abnormalities observed were abnormalities of the telencephalon, mandible or tail, including cleft lip, and craniofacial and forelimb hematomas.

With respect to morphogenesis, no remarkable change was observed in the cultured control (without vehicle) (n = 8) and control (with vehicle) (n = 8) groups after 48 h (Figure 2, Figure 4 and Figure 5). However, slight abnormalities of the telencephalon, mandible or tail, including cleft lip, and craniofacial or forelimb hematomas, were observed in 50% (n = 8/16) of the cultured embryos treated with the low-dose contaminated medium (n = 16) compared with the control (with vehicle) group (Figure 4, Figure 5 and Figure 6 and Supplementary Table S2). Craniofacial abnormalities, including cleft lip, and abnormalities of forelimbs, hindlimbs or tail, and limb hematomas were observed in 100% (n = 17/17) of the embryos treated with the high-dose contaminated medium (n = 17) for 48 h (Figure 4, Figure 5 and Figure 6 and Supplementary Table S2). These results indicate that the organohalogen contaminants extracted from the whale products have teratogenic ability during the period of organogenesis in rat embryos in a dose-dependent manner.

The concentrations of the contaminants (0.3 ppm for PCBs) that interfere with normal embryonic development are within the range of levels measured in the blood of marine mammals [18]. Although the concentrations of PCBs in human blood are about two orders of magnitude lower than those in the culture medium [19,20], the placental transfer of the lipophilic contaminants to the embryo during gestation can result in localized concentrations that interfere with growth and development [21].

## 4. Conclusions

We investigated the direct effects of the organohalogen contaminants, PCBs, DDTs and CHLs, during the critical period of organogenesis using the whole-rat-embryo culture system. The organochlorine contaminants extracted from whale products were readily transferred to rat embryos. Cultured rat embryos exposed to the low-dose contaminants showed slight abnormalities of the telencephalon, mandible or tail, including cleft lip, and craniofacial or forelimb hematomas with an incidence of 50%. All the high-dose exposed embryos developed morphological abnormalities. This study indicates that the organohalogen contaminants extracted from whale products have teratogenic capability at environmentally relevant concentrations during the period of organogenesis in the rat embryo.

## Figures and Tables

**Table 1:** Levels of the organohalogen compounds in the culture medium and embryos and the culture medium to embryo ratio after incubation for 48 h.

	Low-Dose Exposure			High-Dose Exposure		
	Culture Medium	Embryo	Ratio	Culture Medium	Embryo	Ratio
	(ppm on wet weight basis)			(ppm on wet weight basis)		
ΣPCB	0.32 ± 0.06	2.44 ± 0.78	7.6	2.15 ± 0.21	13.4 ± 1.95	6.2
ΣDDT	0.16 ± 0.03	1.48 ± 0.22	9.3	1.99 ± 0.36	15.7 ± 3.12	7.9
ΣCHL	0.02 ± 0.01	0.17 ± 0.02	8.5	0.20 ± 0.03	1.74 ± 0.46	8.7

E11.5 rat embryos were cultured for 48 h at 38 °C by rotating in 100% rat serum. The values are expressed as the mean ± S.D. for four embryos. ΣPCB: sum of 14 PCB isomers, ΣDDT: sum of *p*,*p*′-DDE [1,1-bis(4-chlorophenyl)-2,2-dichloroethylene], *p*,*p*′-DDT [1,1-bis(4-chlorophenyl)-2,2,2-trichloroethane], and *p*,*p*′-DDD [1,1-bis(4-chlorophenyl)-2,2-dichloroethane)], ΣCHL: sum of trans-/cis-nonachlor, trans-/cis-chlordane, and oxychlordane.

**Table 2:** Crown–rump length and total number of somites of rat embryos cultured for 24 and 48 h.

	Crown–Rump Length (mm)		Total Number of Somites (No.)	
	24 h	48 h	24 h	48 h
Control (without vehicle) (8)	5.90 ± 0.13	7.33 ± 0.13	39.2 ± 0.75	44.2 ± 0.3
Control (with vehicle) (8)	5.90 ± 0.13	7.33 ± 0.13	39.2 ± 0.33	44.2 ± 0.3
Low-dose exposure (16)	5.90 ± 0.07	7.20 ± 0.20	39.2 ± 0.75	43.7 ± 0.8
High-dose exposure (17)	5.96 ± 0.13	6.73 ± 0.15 *	38.8 ± 0.33	40.6 ± 0.8 *

E11.5 rat embryos were cultured for 48 h. The values are expressed as the mean ± S.D. for 8–17 embryos. Number of embryos were showed in parenthesis. * *p* < 0.05, significantly different from the vehicle control.

**Table 3:** Yolk sac and embryonic body circulation scores of rat embryos cultured for 24 and 48 h.

	Yolk Sac Circulation Score		Embryonic Body Circulation Score	
	24 h	48 h	24 h	48 h
Control (without vehicle) (8)	5.00 ± 0.03	5.00 ± 0.06	5.00 ± 0.03	5.00 ± 0.06
Control (with vehicle) (8)	5.00 ± 0.03	5.00 ± 0.06	5.00 ± 0.03	5.00 ± 0.06
Low-dose exposure (16)	5.00 ± 0.06	4.81 ± 0.06	5.00 ± 0.06	4.88 ± 0.06
High-dose exposure (17)	5.00 ± 0.06	4.75 ± 0.13	5.00 ± 0.06	4.81 ± 0.06

E11.5 rat embryos were cultured for 48 h. The values are expressed as the mean ± S.D. for four embryos. Number of embryos were showed in parenthesis.

## Data Availability

Not applicable.

## Supplementary Materials

The following supporting information can be downloaded at: https://www.mdpi.com/article/10.3390/ijerph191912065/s1, Figure S1: Rotator apparatus for whole-embryo cultures and cultured embryos. Table S1: Concentrations of all PCB congeners and pesticides in whale bacon product purchased in 2000, Japan. Table S2: Summary of morphogenic abnormalities observed in rat embryos cultured for 48 h with low- and high-dose organohalogen contaminants from whale bacon.
