# Supplementary material for: Teratogenic Effects of Organohalogen Contaminants Extracted from Whale Bacon in a Whole-Rat-Embryo Culture System
Source: Int J Environ Res Public Health. 2022 Sep 23;19(19):12065. doi: 10.3390/ijerph191912065 (PMC9566164; doi:10.3390/ijerph191912065)
Supplement: Supplementary file 1 [file ijerph-19-12065-s001.zip › ijerph-1901336-supplementary.pdf]

Supplementary Table S1. Concentrations of all PCB congeners and pesticides in whale bacon product purchased in 2000, Japan.

IUPAC	PCB congeners	Concentration (ng/g lipid)
28	2,4,4'-triCB	4.1
44	2,2',3,5'-tetraCB	7.1
47	2,2',4,4'-tetraCB	11.0
52	2,2',5,5'-tetraCB	30.6
60	2,3,4,4'-tetraCB	6.2
66	2,3',4,4'-tetraCB	20.9
70	2,3',4',5-tetraCB	4.2
74	2,4,4',5-tetraCB	19.3
85	2,2',3,4,4'-pentaCB	21.0
87	2,2',3,4,5'-pentaCB	26.5
92	2,2',3,5,5'-pentaCB	23.8
95	2,2',3,5',6-pentaCB	53.2
97	2,2',3',4,5-pentaCB	18.4
99	2,2',4,4',5-pentaCB	99.6
101	2,2',4,5,5'-pentaCB	111.5
105	2,3,3',4,4'-pentaCB	61.0
107	2,3,3',4',5-pentaCB	10.2
110	2,3,3',4',6-pentaCB	14.6
114	2,3,4,4',5-pentaCB	6.8
118	2,3',4,4',5-pentaCB	173.7
123	2',3,4,4',5-pentaCB	3.3
128	2,2',3,3',4,4'-hexaCB	102.0
130	2,2',3,3',4,5'-hexaCB	21.1
132	2,2',3,3',4,6'-hexaCB	46.6
133	2,2',3,3'5,5'-hexaCB	16.7
135	2,2',3,3',5,6'-hexaCB	25.7
137	2,2',3,4,4',5-hexaCB	25.6
138	2,2',3,4,4',5'-hexaCB	389.2
139	2,2',3,4,4',6-hexaCB	131.6
141	2,2',3,4,5,5'-hexaCB	18.7
144	2,2',3,4,5',6-hexaCB	14.8
146	2,2',3,4',5,5'-hexaCB	64.3

147	2,2',3,4',5,6-hexaCB	7.6
151	2,2',3,5,5',6-hexaCB	47.9
153	2,2',4,4',5,5'-hexaCB	526.2
156	2,3,3',4,4',5-hexaCB	29.7
157	2,3,3',4,4',5'-hexaCB	13.4
158	2,3,3',4,4',6-hexaCB	21.5
164	2,3,3',4',5',6-hexaCB	78.2
166	2,3,4,4',5,6-hexaCB	9.7
167	2,3',4,4',5,5'-hexaCB	22.6
170	2,2',3,3',4,4',5-heptaCB	115.9
171	2,2',3,3',4,4',6-heptaCB	21.3
172	2,2',3,3',4,5,5'-heptaCB	15.5
174	2,2',3,3',4,5,6'-heptaCB	60.5
175	2,2',3,3',4,5',6-heptaCB	5.6
177	2,2',3,3',4',5,6-heptaCB	42.0
178	2,2',3,3',5,5',6-heptaCB	25.9
179	2,2',3,3',5,6,6'-heptaCB	30.2
180	2,2',3,4,4',5,5'-heptaCB	244.6
182	2,2',3,4,4',5,6'-heptaCB	119.2
183	2,2',3,4,4',5',6-heptaCB	68.2
189	2,3,3',4,4',5,5'-heptaCB	11.0
190	2,3,3',4,4',5,6-heptaCB	0
191	2,3,3',4,4',5',6-heptaCB	4.4
194	2,2',3,3',4,4',5,5'-octaCB	43.8
195	2,2',3,3',4,4',5,6-octaCB	7.2
196	2,2',3,3',4,4',5',6-octaCB	0
198	2,2',3,3',4,5,5',6-octaCB	2.5
200	2,2',3,3',4,5',6,6'-octaCB	5.1
202	2,2',3,3'5,5',6,6'-octaCB	8.5
203	2,2',3,4,4',5,5',6-octaCB	29.1
206	2,2',3,3',4,4',5,5',6-nonaCB	11.1
208	2,2',3,3',4,5,5',6,6'-nonaCB	4.2
209	2,2',3,3',4,4',5,5',6,6'-decaCB	10.1
	total	3226.0

	Pesticide	
	β-HCH	74.2
	γ-HCH	3.5
	heptachlor epoxide	6.3
	dieldrin	20.2
	p,p'-DDE	1087.7
	p,p'-DDD	256.8
	p,p'-DDT	228.2
	trans-chlordane	4.9
	cis-chlordane	69.0
	trans-nonachlor	142.0
	cis-nonachlor	82.4
	oxychlordane	6.6
	total	1982.0

GC/MS system (GC-17A, QP5000, Shimadzu, Japan) with a DB-5 capillary column (60 m × 0.25 mm, i.d.), Temperature program: 100℃, 2 min, 100-250℃ at 20℃/min, 250-280℃ at 2℃/min (Mimura *et al*., 1999).

Supplementary Table S2. Summary of morphogenic abnormalities observed in rat embryos cultured for 48 h with low- and high-dose organohalogen contaminants from whale bacon

	Control (without vehicle)	Control (with vehicle)	Low-dose exposure	High-dose exposure
Number of embryos cultured	8	8	16	17
Number of morphogenic abnormalities	0	0	8	17
Morphogenic abnormalities				
Microcephaly	0	0	0	10
Abnormalities of the telencephalon or mandible, Maxillary hypoplasia	0	0	3	10
Cleft lip	0	0	3	7
Edema	0	0	2	2
Hematoma	0	0	3	2
Internal hemorrhage	0	0	2	4
Kinked tail	0	0	5	5
Short tail	0	0	2	10

Multiple morphological abnormalities may be observed in one individual rat embryos cultured.

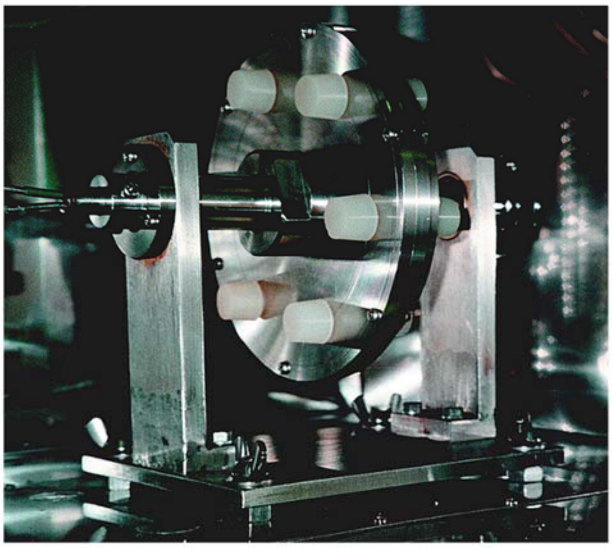

Whole-embryo culture system

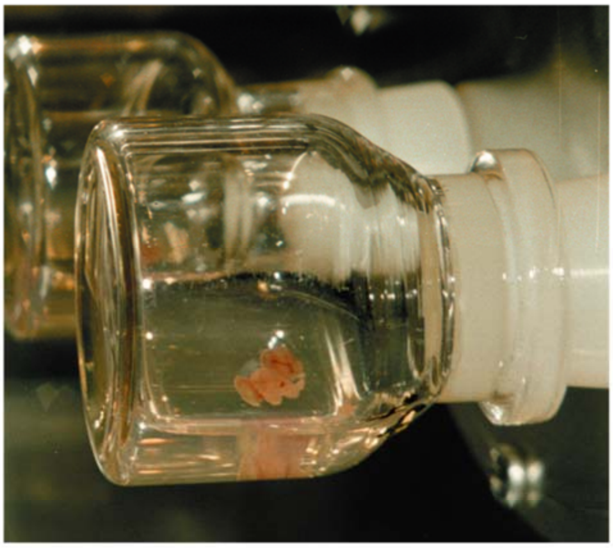

Cultured embryos

Supplementary Figure S1. Rotator apparatus for whole-embryo cultures and cultured embryos
